# Association between dietary antioxidants intake and childhood eczema: results from the NHANES database

**DOI:** 10.1186/s41043-024-00501-x

**Published:** 2024-01-18

**Authors:** Jing Xu, Hongxin Li

**Affiliations:** Department of Dermatology, Children’s Hospital Affiliated to Capital Institute of Pediatrics, Beijing, 100020 People’s Republic of China

**Keywords:** Dietary antioxidants, Eczema, NHANES, Children and adolescents

## Abstract

**Background:**

High dietary intake of antioxidants reduces the risk of allergic sensitization in children. However, there was no sufficient evidence for the effect of dietary antioxidants intake on childhood eczema. Herein, this study aimed to explore the roles of different dietary antioxidants in childhood eczema.

**Methods:**

Data of 2305 children and adolescents aged < 18 years old were extracted from the National Health and Nutrition Examination Survey database in 2005–2006 in this cross-sectional study. The associations between dietary antioxidants intake and childhood eczema were explored using univariate and multivariate logistic regression analyses, with odds ratios (ORs) and confidence intervals (CIs). Subgroup analyses based on age and gender were also performed.

**Results:**

A total of 268 (11.6%) children had eczema. After adjusting for covariates, we found no significant associations between dietary intake of β-carotene, vitamin C, selenium (Se), and retinol and childhood eczema. However, compared with children and adolescents whose dietary zinc (Zn) intake < 7.47 mg, those who had dietary Zn intake level ≥ 11.83 mg seemed to have lower odds of eczema [OR 0.45, 95% CI 0.28–0.73]. In addition, subgroup analysis showed that especially in children and adolescents aged 1–11 years old, whatever the gender, a higher dietary intake level of Zn may benefit childhood eczema (all *P* < 0.05).

**Conclusion:**

We concluded dietary Zn intake was negatively associated with childhood eczema. Further studies are needed to explore the roles of dietary antioxidants intake in childhood eczema.

**Supplementary Information:**

The online version contains supplementary material available at 10.1186/s41043-024-00501-x.

## Background

Eczema is a chronic relapsing skin inflammatory disease characterized by defective skin barrier function [1]. The global prevalence of childhood eczema is around 20.0% [2]. The early eczema-related comorbidities increase the risk of future persistent multimorbidity [3]. Results of previous longitudinal birth cohort studies indicated that eczema in early life was associated with up to sevenfold increased risks of asthma and allergy in later life, compared with the never eczema phenotype [4, 5]. Therefore, identification of factors affecting early eczema development is vital to reduce the prevalence of eczema and the overall disease burden.

Eczema as a disease of immunological interest is linked to changes in nutrition along with general environmental changes, especially during a critical period of life [6]. Oxidative stress (OS) is one of the most important factors in eczema pathophysiology. OS is the result of reactive oxygen species (ROS) overproduction or of inadequate antioxidant defense mechanisms, leading to disruption of the oxidant–antioxidant balance toward the oxidants [7]. OS can disrupt barrier function, enhance proinflammatory cytokine production and activate naive T cells and cellular dermal infiltration, and consequently aggravate eczema lesions [8, 9]. Dietary antioxidants, such as vitamin C, β-carotene, vitamin E, selenium (Se), zinc (Zn), and retinol, take part in redox reactions and act by scavenging reactive oxygen-free radicals in order to remit inflammation [10, 11]. A diet low in antioxidants may increase susceptibility to oxidant injury and inflammation [12]. A previous study has reported that increased dietary intakes of Zn was associated with a low risk of atopic eczema (AE) in children [6]. Patel et al. [13] also showed that in children aged 5–8 years old, increased dietary intake of β-carotene was related to a decreased risk of allergic sensitization and serum IgE levels.

Recent studies have not shown sufficient evidences for the effect of dietary intake of Zn, β-carotene and other dietary antioxidants on the risk of childhood eczema. Herein, this study aims to explore the associations between different dietary antioxidants intake and childhood eczema.

## Methods

### Study design and population

Data of participants in this cross-sectional study were extracted from the National Health and Nutrition Examination Survey (NHANES) in 2005–2006. NHANES is a representative survey research program to assess the health and nutritional status of American adults and children. Regular data collection of approximately 5,000 persons is carried out from 15 areas since 1999 and examines in two-year periods. NHANES 2005–2006 is the only cycle with complete questionnaires on eczema. More details of statistical data can be found on the NHANES website [14].

A total of 4,785 children and adolescents (aged < 18 years old) in the NHANES were initially included. The exclusion criteria were (1) missing information of dietary intake of vitamin E, β-carotene, vitamin C, Zn, Se or retinol, and (2) children having an answer of ‘refused,’ ‘don’t know’ or ‘missing’ for the eczema questionnaire. Finally, 2,305 of them were eligible. The NHANES survey is approved by the institutional review board (IRB) of the National Center for Health Statistics (NCHS). The participants’ legal guardians/next of kin have provided written informed consent for participation. Since all the data were de-identified and publicly available, no ethical approval of this study by the IRB of Children’s Hospital Affiliated to Capital Institute of Pediatrics was required. In addition, all study methods were carried out in accordance with relevant guidelines and regulations (declaration of Helsinki).

### Measurement of dietary antioxidants intake

In NHANES, dietary antioxidants intake was collected via two 24-h dietary recalls, in which participants reported individual foods and drinks consumed during the midnight-to-midnight 24-h period prior to the in-person dietary interview. Participants under the age of 16 years old were interviewed by a proxy interviewee (typically their parents). The first dietary recall interview is collected in-person in the Mobile Examination Center (MEC), and the second interview is collected by telephone 3 to 10 days later. NHANES conducted the coding of interview data and conversion to total nutrient intakes by using the United States Department of Agriculture (USDA) Food and Nutrient Database for Dietary Studies, 5.0 (FNDDS 5.0) [15].

Data of dietary antioxidants intake including vitamin E, β-carotene, vitamin C, Zn, Se and retinol were extracted in this study. We divided the antioxidants consumption into three levels respectively according to their own quantiles: vitamin E (< 3.82, 3.82 ≤ vitamin E < 6.28, and ≥ 6.28 mg), β-carotene (< 230, 230 ≤ β-carotene < 613, and ≥ 613 mcg), vitamin C (< 35.5, 35.5 ≤ vitamin C < 96.1, and ≥ 96.1 mg), Zn (< 7.47, 7.47 ≤ Zn < 11.83, and ≥ 11.83 mg), Se (< 68.8, 68.8 ≤ Se < 104.4, and ≥ 104.4 mcg) and retinol (< 253, 253 ≤ retinol < 500, and ≥ 500 mcg).

### Diagnosis of eczema

The diagnosis of childhood eczema was according to the self-reported NHANES questionnaire. Respondents for the interviews included the following: a proxy for child aged < 6 years old; a proxy with the assistance of the child for those aged 6–8 years old; assistance of a proxy for child aged 9–11 years old; and children aged ≥ 12 years old who answered by themselves. Children and adolescents who had self-reported symptoms (an itchy rash, which was intermittently coming and going) in the last 12 months or a positive answer to the question “Has a doctor or other health professional ever told you that you have eczema?” were recognized as childhood eczema [16].

### Covariates selection

We collected variables from the NHANES database including age, gender, race, family educational background, poverty-to-income ratio (PIR), exposure to environmental tobacco smoke, insurance, maternal age, asthma, hay fever, food allergy, peanut allergy, egg allergy, milk allergy, shrimp allergy, height, weight, body mass index (BMI), cotinine, C-reactive protein (CRP), vitamin D, energy intake, polyunsaturated fatty acids (PUFA) intake, and serum immunoglobulin E (IgE) level.

The diagnoses of asthma and hay fever were both according to the NHANES questionnaires similar to that of the eczema. A positive response to both questions: “Has a doctor or other health professional ever told you that you have asthma?” and “In the past 12 months (have you/he/she) had wheezing or whistling in (your/his/her) chest?” were used to recognize asthma. Hay fever (itchy, runny, or blocked nose without a cold accompanied by red itchy eyes) was diagnosed according to the positive response for the question: “Have you had hay fever in the past 12 months?” [17]. The sensitization to common food allergens in the USA, including peanut allergy, egg allergy, milk allergy, and shrimp allergy, was assessed using serum concentrations of allergen specific IgE according to a previous study [18]. Serum samples were collected for the analyses by the Pharmacia Diagnostics Immuno CAP 1000 System (Pharmacia Diagnostics, Kalamazoo, MI, USA) [19].

### Statistical analyses

Normal distribution data were expressed as mean ± standard deviation (Mean ± SD), and t test was used for comparison between groups. Skewed distribution data were expressed as median and quartiles [M (Q1, Q3)], and used rank sum test for comparison. Categorical data were expressed as frequency and constituent ratio [*N* (%)], and chi-square test (*χ*^2^) was used for comparison.

Univariate logistic regression analysis was used for covariates screening. Univariate and multivariate logistic regression analyses were used to explore the association between dietary antioxidants intake and childhood eczema. Model 1 was the crude model. Model 2 adjusted for age, gender, race, family educational background, PIR, insurance, asthma, hay fever, food allergy, BMI, energy intake, PUFA intake and serum IgE level. Model 3 adjusted for variables included in the Model 2 and different dietary antioxidants. Subgroup analyses of age and gender were also performed.

The evaluation index was odds ratios (ORs) with 95% confidence intervals (CIs). Two-sided* P* < 0.05 was considered significant. To eliminate differences results from dimension, we normalized the data [with the formula: (x-mean)/standard deviation], and the normalization can magnify the influencing of these variables on the outcome to make it more direct. Statistical analyses were performed using SAS 9.4 (SAS Institute, Cary, NC, USA). Missing data were deleted (accounting for more than 20%) or interpolated using the multiple interpolation method (accounting for ≤ 20%). The sensitive analysis of characteristics of participants before and after interpolation of missing variables is shown in Additional file 1: Table S2.

## Results

### Characteristics of eligible children

There are 4,785 children and adolescents aged < 18 years old in the NHANES database in 2005–2006. Those who without information of eczema (*n *= 536), Zn intake (*n *= 816), serum IgE level (*n *= 571), cotinine (*n *= 454), family educational background (*n *= 84), exposure to environmental tobacco smoke (*n *= 8), insurance (*n *= 9), and hay fever (*n *= 3) were excluded. Finally, 2,304 of them were eligible.

Table 1 showed the characteristics of participants in non-eczema group and eczema group. Among the eligible children and adolescents, 268 (11.6%) children had eczema. The average age of the study population was 12 years old, and there were 1175 (51.0%) females. Among total population, 384 (16.7%) had asthma, 86 (3.7%) had hay fever, and 593 (25.7%) had food allergy. The median dietary intakes of Zn (9.62 mg vs. 8.49 mg) and Se (87.75 mcg vs. 79.80 mcg) were significantly different between non-eczema group and eczema group. In addition, race, family educational background, PIR, insurance, peanut allergy, egg allergy, height, weight, BMI, and serum IgE level were all different between these two groups (all *P *< 0.05).

**Table 1:** Characteristics of eligible children and adolescents

Variables	Total (*n *= 2304)	Non-eczema (*n *= 2036)	Eczema (*n *= 268)	*P*
Age, years, *M* (Q_1_, Q_3_)	12.00 (8.00, 15.00)	12.00 (8.00, 15.00)	11.00 (6.00, 14.00)	< 0.001
Age, years, *n* (%)				0.003
1–5	312 (13.5)	262 (12.9)	50 (18.7)	
6–11	738 (32.0)	642 (31.5)	96 (35.8)	
12–17	1254 (54.4)	1132 (55.6)	122 (45.5)	
Gender, *n* (%)				0.633
Female	1175 (51.0)	1042 (51.2)	133 (49.6)	
Male	1129 (49.0)	994 (48.8)	135 (50.4)	
Race, *n* (%)				< 0.001
Mexican American	750 (32.6)	721 (35.4)	29 (10.8)	
Other Hispanic	79 (3.4)	67 (3.3)	12 (4.5)	
Non-Hispanic White	651 (28.3)	560 (27.5)	91 (34.0)	
Non-Hispanic Black	692 (30.0)	572 (28.1)	120 (44.8)	
Other races	132 (5.7)	116 (5.7)	16 (6.0)	
Family educational background, *n* (%)				< 0.001
Less than 9th grade	272 (11.8)	266 (13.1)	6 (2.2)	
9–11th grade	432 (18.8)	396 (19.5)	36 (13.4)	
High school grade	539 (23.4)	479 (23.5)	60 (22.4)	
Some college	646 (28.0)	560 (27.5)	86 (32.1)	
College graduate	415 (18.0)	335 (16.5)	80 (29.9)	
PIR, *M* (Q_1_, Q_3_)	1.81 (0.95, 3.48)	1.71 (0.93, 3.36)	2.55 (1.20, 4.12)	< 0.001
Exposure to environmental tobacco smoke, *n* (%)				0.968
No	1941 (84.2)	1715 (84.2)	226 (84.3)	
Yes	363 (15.8)	321 (15.8)	42 (15.7)	
Insurance, *n* (%)				< 0.001
No	334 (14.5)	318 (15.6)	16 (6.0)	
Yes	1970 (85.5)	1718 (84.4)	252 (94.0)	
Maternal age, years, Mean ± SD	25.90 ± 6.00	25.84 ± 6.03	26.37 ± 5.79	0.174
Asthma, *n* (%)				< 0.001
No	1920 (83.3)	1730 (85.0)	190 (70.9)	
Yes	384 (16.7)	306 (15.0)	78 (29.1)	
Hay fever, *n* (%)				< 0.001
No	2218 (96.3)	1974 (97.0)	244 (91.0)	
Yes	86 (3.7)	62 (3.1)	24 (9.0)	
Food allergy, *n* (%)				0.003
No	1711 (74.3)	1532 (75.3)	179 (66.8)	
Yes	593 (25.7)	504 (24.8)	89 (33.2)	
Peanut allergy, *n* (%)				< 0.001
No	2045 (88.8)	1829 (89.8)	216 (80.6)	
Yes	259 (11.2)	207 (10.2)	52 (19.4)	
Egg allergy, *n* (%)				< 0.001
No	2176 (94.4)	1937 (95.1)	239 (89.2)	
Yes	128 (5.6)	99 (4.9)	29 (10.8)	
Milk allergy, *n* (%)				0.063
No	2031 (88.2)	1804 (88.6)	227 (84.7)	
Yes	273 (11.9)	232 (11.4)	41 (15.3)	
Shrimp allergy, *n* (%)				0.654
No	2147 (93.2)	1899 (93.3)	248 (92.5)	
Yes	157 (6.8)	137 (6.7)	20 (7.5)	
Height, cm, Mean ± SD	147.28 ± 23.04	147.74 ± 22.76	143.78 ± 24.87	0.014
Weight, kg, *M* (Q_1_, Q_3_)	48.05 (28.40, 63.00)	48.55 (29.00, 63.30)	43.70 (24.65, 59.50)	0.003
BMI, kg/m^2^, Mean ± SD	21.10 ± 6.02	21.23 ± 6.09	20.15 ± 5.36	0.003
Cotinine, ng/mL, *M* (Q_1_, Q_3_)	0.05 (0.02, 0.30)	0.05 (0.02, 0.28)	0.04 (0.02, 0.36)	0.653
CRP, mg/dL, *M* (Q_1_, Q_3_)	0.04 (0.01, 0.13)	0.04 (0.01, 0.13)	0.03 (0.01, 0.10)	0.144
Vitamin D, nmol/L, *M* (Q_1_, Q_3_)	58.00 (47.10, 71.30)	58.00 (47.10, 70.10)	58.00 (47.10, 71.30)	0.729
IgE, IU/mL, *M* (Q_1_, Q_3_)	57.05 (21.20, 177.00)	54.55 (20.30, 167.00)	82.20 (26.35, 271.00)	< 0.001
Dietary intake				
Energy, kcal, *M* (Q_1_, Q_3_)	1890.00 (1469.50, 2460.00)	1895.50 (1473.50, 2474.50)	1840.50 (1409.00, 2393.50)	0.139
PUFA, gm, *M* (Q_1_, Q_3_)	13.16 (8.67, 19.47)	13.20 (8.72, 19.61)	12.79 (8.45, 18.54)	0.451
Vitamin E, mg, *M* (Q_1_, Q_3_)	4.92 (3.29, 7.15)	4.99 (3.32, 7.21)	4.64 (3.15, 6.76)	0.089
β-carotene, mcg, *M* (Q_1_, Q _3_)	363.00 (180.50, 839.50)	366.00 (181.00, 848.50)	339.00 (168.00, 787.00)	0.348
Vitamin C, mg, *M* (Q_1_, Q_3_)	60.80 (25.80, 123.30)	61.45 (25.75, 125.00)	53.05 (26.65, 109.40)	0.223
Zinc, mg, *M* (Q_1_, Q_3_)	9.49 (6.58, 13.80)	9.62 (6.63, 14.05)	8.49 (6.19, 11.66)	0.001
Selenium, mcg, *M* (Q_1_, Q_3_)	87.10 (59.35, 119.20)	87.75 (60.85, 120.00)	79.80 (52.80, 112.40)	0.018
Retinol, mcg, *M* (Q_1_, Q_3_)	366.00 (201.00, 603.00)	365.50 (202.00, 608.50)	378.50 (179.50, 568.00)	0.769
Vitamin E, mg, *n* (%)				0.461
< 3.82	757 (32.9)	660 (32.4)	97 (36.2)	
[3.82, 6.28)	762 (33.1)	677 (33.3)	85 (31.7)	
≥ 6.28	785 (34.1)	699 (34.3)	86 (32.1)	
β-carotene, mcg, *n* (%)				0.296
< 230	757 (32.9)	658 (32.3)	99 (36.9)	
[230, 613)	763 (33.1)	682 (33.5)	81 (30.2)	
≥ 613	784 (34.0)	696 (34.2)	88 (32.8)	
Vitamin C, mg, *n* (%)				0.489
< 35.5	758 (32.9)	672 (33.0)	86 (32.1)	
[35.5, 96.1)	762 (33.1)	665 (32.7)	97 (36.2)	
≥ 96.1	784 (34.0)	699 (34.3)	85 (31.7)	
Zinc, mg, *n* (%)				< 0.001
< 7.47	759 (32.9)	649 (31.9)	110 (41.0)	
[7.47, 11.83)	761 (33.0)	669 (32.9)	92 (34.3)	
≥ 11.83	784 (34.0)	718 (35.3)	66 (24.6)	
Selenium, mcg, *n* (%)				0.114
< 68.8	759 (32.9)	656 (32.2)	103 (38.4)	
[68.8, 104.4)	760 (33.0)	676 (33.2)	84 (31.3)	
≥ 104.4	785 (34.1)	704 (34.6)	81 (30.2)	
Retinol, mcg, *n* (%)				0.722
< 253	757 (32.9)	667 (32.8)	90 (33.6)	
[253, 500)	763 (33.1)	680 (33.4)	83 (31.0)	
≥ 500	784 (34.0)	689 (33.8)	95 (35.5)	

*PIR* Poverty income ratio, *BMI* Body mass index, *CRP* C-reactive protein, *PUFA* Polyunsaturated fatty acid, *IgE* Immunoglobulin E

Statistics: t test, rank sum test and chi-square test

### Association between dietary antioxidants intake and childhood eczema

We first screened the covariates associated with childhood eczema (Additional file 1: Table S1). The results showed that age, race, family educational background, PIR, insurance, asthma, hay fever, food allergy, BMI, and serum IgE level were associated with childhood eczema (all *P *< 0.05). In addition, gender, energy intake, and PUFA intake have all been reported to be associated with allergic diseases, so that they were all included in the adjusted models [20–22].

Then we explored the relationship between different antioxidants intake and childhood eczema (Table 2). After adjusting for the above covariates, we found no significant association of dietary intakes of β-carotene, vitamin C, Se, and retinol with eczema (*P* > 0.05). Dietary intakes of vitamin E [OR 0.95, 95% CI 0.90–0.99] and Zn [OR 0.96, 95% CI 0.93–0.99] were both negatively associated with eczema. However, when further adjusted for these antioxidants to each other, we only found the negative relationship between dietary Zn intake and eczema [OR 0.96, 95% CI 0.93–0.99]. Compared with children and adolescents whose dietary intake of Zn < 7.47 mg, those who had dietary Zn intake level ≥ 11.83 mg seemed to have lower odds of eczema [OR 0.45, 95% CI 0.28–0.73].

**Table 2:** Association between dietary antioxidants intake and childhood eczema

Variables	Model 1		Model 2		Model 3	
	OR (95% CI)	*P*	OR (95% CI)	*P*	OR (95% CI)	*P*
*Original data*						
Vitamin E (mg)	0.96 (0.93–0.99)	0.037	0.95 (0.90–0.99)	0.048	0.96 (0.91–1.01)	0.109
β-carotene (mcg)	1.00 (1.00–1.00)	0.713	1.00 (1.00–1.00)	0.530	1.00 (1.00–1.00)	0.206
Vitamin C (mg)	1.00 (1.00–1.00)	0.051	1.00 (1.00–1.00)	0.181	1.00 (1.00–1.00)	0.203
Zinc (mg)	0.97 (0.95–0.99)	0.003	0.96 (0.93–0.99)	0.018	0.96 (0.93–0.99)	0.027
Selenium (mcg)	1.00 (1.00–1.00)	0.072	1.00 (1.00–1.00)	0.779	1.00 (1.00–1.00)	0.838
Retinol (mcg)	1.00 (1.00–1.00)	0.401	1.00 (1.00–1.00)	0.591	1.00 (1.00–1.00)	0.777
*Normalized data*						
Vitamin E (mg)	0.84 (0.71–0.99)	0.037	0.77 (0.60–0.99)	0.048	0.80 (0.61–1.05)	0.109
β-carotene (mcg)	1.02 (0.91–1.15)	0.713	1.04 (0.92–1.18)	0.530	1.08 (0.96–1.23)	0.206
Vitamin C (mg)	0.87 (0.75–1.00)	0.051	0.89 (0.76–1.05)	0.181	0.89 (0.75–1.06)	0.203
Zinc (mg)	0.80 (0.68–0.93)	0.003	0.77 (0.61–0.95)	0.018	0.76 (0.60–0.97)	0.027
Selenium (mcg)	0.88 (0.76–1.01)	0.072	0.97 (0.77–1.22)	0.779	1.03 (0.81–1.30)	0.838
Retinol (mcg)	0.94 (0.83–1.08)	0.401	0.96 (0.81–1.12)	0.591	1.03 (0.86–1.22)	0.777
*Vitamin E*						
< 3.82	Ref		Ref		Ref	
[3.82, 6.28)	0.85 (0.63–1.17)	0.320	0.88 (0.63–1.24)	0.474	0.93 (0.65–1.32)	0.675
≥ 6.28	0.84 (0.61–1.14)	0.260	0.93 (0.61–1.42)	0.740	1.01 (0.64–1.59)	0.960
*P* for trend		0.258		0.703		0.938
*β-carotene*						
< 230	Ref		Ref		Ref	
[230, 613)	0.79 (0.58–1.08)	0.138	0.80 (0.57–1.12)	0.195	0.82 (0.58–1.16)	0.257
≥ 613	0.84 (0.62–1.14)	0.266	0.89 (0.64–1.25)	0.512	0.91 (0.63–1.31)	0.606
*P* for trend		0.261		0.526		0.652
*Vitamin C*						
< 35.5	Ref		Ref		Ref	
[35.5, 96.1)	1.14 (0.84–1.55)	0.407	1.08 (0.78–1.50)	0.646	1.11 (0.79–1.55)	0.556
≥ 96.1	0.95 (0.69–1.31)	0.753	0.96 (0.67–1.36)	0.808	0.95 (0.65–1.39)	0.810
*P* for trend		0.749		0.812		0.790
*Zinc*						
< 7.47	Ref		Ref		Ref	
[7.47, 11.83)	0.81 (0.60–1.09)	0.168	0.76 (0.54–1.05)	0.097	0.74 (0.52–1.06)	0.104
≥ 11.83	0.54 (0.39–0.75)	< 0.001	0.50 (0.33–0.78)	0.002	0.45 (0.28–0.73)	0.001
*P* for trend		< 0.001		0.002		0.001
*Selenium*						
< 68.8	Ref		Ref		Ref	
[68.8, 104.4)	0.79 (0.58–1.08)	0.136	0.81 (0.58–1.14)	0.223	0.91 (0.64–1.30)	0.616
≥ 104.4	0.73 (0.54–0.99)	0.049	0.89 (0.58–1.35)	0.578	1.11 (0.71–1.76)	0.641
*P* for trend		0.047		0.512		0.796
*Retinol*						
< 253	Ref		Ref		Ref	
[253, 500)	0.90 (0.66–1.24)	0.535	0.85 (0.60–1.19)	0.343	0.91 (0.64–1.29)	0.578
≥ 500	1.02 (0.75–1.39)	0.890	1.05 (0.73–1.52)	0.788	1.25 (0.85–1.85)	0.253
*P* for trend		0.883		0.786		0.273

*OR* Odds ratio, *CI* Confidence interval, *Ref* Reference, *Model 1* Univariate logistic regression, *Model 2* Adjusted for age, gender, race, family educational background, PIR, insurance, asthma, hay fever, food allergy, BMI, energy intake, PUFA intake and IgE, *Model 3* Vitamin E: adjusted for variables in Model 2 and β-carotene, vitamin C, zinc, selenium, and retinol, *β-carotene* Adjusted for variables in Model 2 and vitamin E, vitamin C, zinc, selenium, and retinol, *Vitamin C* Adjusted for variables in Model 2 and vitamin E, β-carotene, zinc, selenium, and retinol, *Zinc* Adjusted for variables in Model 2 and vitamin E, β-carotene, vitamin C, selenium, and retinol, *Selenium* Adjusted for variables in Model 2 and vitamin E, β-carotene, vitamin C, zinc, and retinol, *Retinol* Adjusted for variables in Model 2 and vitamin E, β-carotene, vitamin C, zinc, and selenium

### Relationships between antioxidants and childhood eczema in age and gender subgroups

We further explored these associations in age (Table 3) and gender subgroups. In children aged 1–5 years old, dietary Zn intake was negatively associated with eczema [OR 0.87, 95% CI 0.76–0.98], while that of β-carotene [OR 1.01, 95% CI 1.01–1.01] and retinol [OR 1.01, 95% CI 1.01–1.01] were both positively associated with eczema. Among children aged 1–11 years old, dietary Zn intake level ≥ 7.47 mg was significantly associated with lower odds of eczema, compared with dietary Zn intake level < 7.47 mg (all *P* < 0.05). Besides, compared with dietary Zn intake level < 7.47 mg, that ≥ 11.83 mg was linked to lower odds of eczema in both male [OR 0.51, 95% CI 0.26–0.99] and female [OR 0.41, 95% CI 0.21–0.82] children and adolescents.

**Table 3:** Association between dietary antioxidants intake and childhood eczema in age subgroups

Variables	1–5 years old (*n *= 312)		6–11 years old (*n *= 738)		12–17 years old (*n *= 1254)		*P* for interaction
	OR (95% CI)	*P*	OR (95% CI)	*P*	OR (95% CI)	*P*	
*Original data*							
Vitamin E (mg)	0.97 (0.83–1.14)	0.753	0.95 (0.85–1.06)	0.337	0.95 (0.88–1.02)	0.154	0.979
β-carotene (mcg)	1.01 (1.01–1.01)	0.003	1.00 (1.00–1.00)	0.447	1.00 (1.00–1.00)	0.767	0.150
Vitamin C (mg)	1.00 (0.99–1.01)	0.704	1.00 (0.99–1.00)	0.201	1.00 (1.00–1.00)	0.702	0.633
Zinc (mg)	0.87 (0.76–0.98)	0.027	0.94 (0.88–1.01)	0.091	0.97 (0.93–1.02)	0.216	0.148
Selenium (mcg)	1.00 (0.98–1.01)	0.792	1.00 (0.99–1.01)	0.615	1.00 (1.00–1.01)	0.578	0.527
Retinol (mcg)	1.01 (1.01–1.01)	0.049	1.00 (1.00–1.00)	0.285	1.00 (1.00–1.00)	0.251	0.412
*Normalized data*							
Vitamin E (mg)	0.88 (0.41–1.92)	0.753	0.76 (0.44–1.32)	0.337	0.77 (0.54–1.10)	0.154	
β-carotene (mcg)	2.12 (1.30–3.47)	0.003	1.09 (0.87–1.36)	0.447	1.03 (0.85–1.24)	0.767	
Vitamin C (mg)	0.89 (0.50–1.59)	0.704	0.80 (0.58–1.12)	0.201	0.96 (0.77–1.19)	0.702	
Zinc (mg)	0.38 (0.16–0.90)	0.027	0.66 (0.41–1.07)	0.091	0.83 (0.62–1.11)	0.216	
Selenium (mcg)	0.89 (0.39–2.05)	0.792	0.89 (0.56–1.41)	0.615	1.09 (0.80–1.48)	0.578	
Retinol (mcg)	1.79 (1.01–3.21)	0.049	0.84 (0.60–1.16)	0.285	1.14 (0.91–1.43)	0.251	
*Vitamin E*							
< 3.82	Ref		Ref		Ref		
[3.82, 6.28)	0.45 (0.15–1.34)	0.151	0.91 (0.47–1.73)	0.766	1.04 (0.61–1.79)	0.876	
≥ 6.28	0.52 (0.10–2.67)	0.431	1.73 (0.76–3.94)	0.192	0.86 (0.45–1.67)	0.664	
*P* for trend		0.442		0.254		0.818	
*β-carotene*							
< 230	Ref		Ref		Ref		
[230, 613)	1.20 (0.48–2.98)	0.697	0.56 (0.30–1.06)	0.073	0.81 (0.48–1.36)	0.422	
≥ 613	1.56 (0.62–3.93)	0.342	0.56 (0.29–1.10)	0.091	0.98 (0.57–1.70)	0.956	
*P* for trend		0.224		0.101		0.864	
*Vitamin C*							
< 35.5	Ref		Ref		Ref		
[35.5, 96.1)	0.99 (0.37–2.68)	0.990	1.04 (0.58–1.87)	0.892	1.32 (0.81–2.17)	0.266	
≥ 96.1	1.49 (0.53–4.21)	0.455	0.90 (0.46–1.77)	0.756	0.89 (0.51–1.55)	0.674	
*P* for trend		0.416		0.825		0.701	
*Zinc*							
< 7.47	Ref		Ref		Ref		
[7.47, 11.83)	0.31 (0.11–0.86)	0.024	0.45 (0.25–0.83)	0.011	1.34 (0.77–2.32)	0.305	
≥ 11.83	0.15 (0.04–0.68)	0.013	0.34 (0.16–0.74)	0.007	0.64 (0.31–1.31)	0.226	
*P* for trend		0.004		0.012		0.228	
*Selenium*							
< 68.8	Ref		Ref		Ref		
[68.8, 104.4)	0.76 (0.29–2.02)	0.588	0.93 (0.51–1.67)	0.803	0.91 (0.52–1.60)	0.751	
≥ 104.4	1.30 (0.31–5.45)	0.722	0.67 (0.30–1.48)	0.325	1.38 (0.71–2.67)	0.345	
*P* for trend		0.868		0.200		0.283	
*Retinol*							
< 253	Ref		Ref		Ref		
[253, 500)	0.84 (0.32–2.18)	0.714	0.82 (0.44–1.55)	0.547	1.18 (0.72–1.95)	0.511	
≥ 500	1.91 (0.66–5.52)	0.234	1.31 (0.68–2.55)	0.422	1.36 (0.77–2.39)	0.293	
*P* for trend		0.294		0.406		0.318	

*OR* Odds ratio, *CI* Confidence interval, *Ref* Reference, *Vitamin E* adjusted for gender, race, family educational background, PIR, insurance, asthma, hay fever, food allergy, BMI, energy intake, PUFA intake, IgE, β-carotene, vitamin C, zinc, selenium, and retinol, *β-carotene* adjusted for gender, race, family educational background, PIR, insurance, asthma, hay fever, food allergy, BMI, energy intake, PUFA intake, IgE, vitamin E, vitamin C, zinc, selenium, and retinol, *Vitamin C* Adjusted for gender, race, family educational background, PIR, insurance, asthma, hay fever, food allergy, BMI, energy intake, PUFA intake, IgE, vitamin E, β-carotene, zinc, selenium, and retinol, *Zinc* Adjusted for gender, race, family educational background, PIR, insurance, asthma, hay fever, food allergy, BMI, energy intake, PUFA intake, IgE, vitamin E, β-carotene, vitamin C, selenium, and retinol, *Selenium* Adjusted for gender, race, family educational background, PIR, insurance, asthma, hay fever, food allergy, BMI, energy intake, PUFA intake, IgE, vitamin E, β-carotene, vitamin C, zinc, and retinol, *Retinol* Adjusted for gender, race, family educational background, PIR, insurance, asthma, hay fever, food allergy, BMI, energy intake, PUFA intake, IgE, vitamin E, β-carotene, vitamin C, zinc and selenium

## Discussion

This study explored the relationships between different dietary antioxidants intake and childhood eczema. Our results showed that higher dietary intake of Zn was associated with lower odds of childhood eczema, and this relationship was also found in children aged 1–11 years old regardless of gender.

The essential micronutrients Zn and Se have complex influences on human immune system and are associated with allergic diseases such as asthma, rhinitis, and atopic dermatitis [23]. A follow-up study from birth to 4 years showed that an increased intake of Zn, with perinatal administration of probiotics, reduced the risk of AE [6]. Topic et al. [24] found that increased Zn- superoxide dismutase (SOD) was associated with eczema in asthmatic children. Another study showed a deficiency of serum Zn (52.0%) and Cu (33.0%) in atopic children characterized by eczema, stomatitis, relapsing herpes, and neurosis [25]. Amin et al. [26] suggested that malonaldehyde (MDA) significantly raised and antioxidants level decreased may be possible causative factors for the pathogenesis of eczema. Similarly, in the current study, the results indicated that higher dietary intake of Zn was associated with lower odds of childhood eczema, and however, we have not found the relationship between dietary Se intake and eczema. Zn is primarily found in animal products and seafood, such as milk and dairy products, meat and meat products [27]. Low fish consumption could explain the insufficient intake of Zn [28]. Also, Zn is a vital nutrient for skin health, which involved in many skin functions, such as the regeneration of skin cells, activation of skin hormone, and control of skin inflammation [29]. In our body, several Zn bound proteins depends on the concentration of Zn for exhibiting their biological activities [30]. Nevertheless, there were some inconsistent results as well on the associations between dietary intake of antioxidant vitamins and minerals and allergic diseases, which discrepancies may be explained by the different roles of nutrients depending on the end-organ manifestation [31, 32]. Therefore, potential mechanisms of dietary Zn intake affecting the childhood eczema needed further exploration.

In subgroup analyses, the association between dietary intake of Zn and childhood eczema was also found in children who aged 1–11 years old. Zn deficiency is highly prevalent in young children (from 6 months to 12 years old) since during periods of rapid growth, they have increased requirements of Zn, which lead to an increased risk of developing Zn deficiency [33]. Similarly, our results supported this opinion, indicating that higher dietary Zn intake may have a potential beneficial effect on eczema in children aged less than 11 years old, which possibly because children during rapid growth may be more sensitive to inflammatory skin disease related to Zn deficiency such as eczema. Besides, with the dietary intake of β-carotene and retinol elevated, the odds of childhood eczema were increased in children aged 1–5 years old. In fact, whether the β-carotene and retinol play risk or preventive roles in allergic disorders is uncertain. A cross-sectional analysis showed no significant difference in activity of retinol in Children with asthma compared with controls [34]. Patel et al. [13] suggested no association between antioxidant intakes and eczema in children aged 5–8 years old. Due to the lack of references on relationships between β-carotene and retinol and eczema in children of 1–5 year of age, our findings need further clarification. Simonsen et al. [35] found that the risk of hand eczema was significantly associated with female gender. Although gender difference has been reported to be related to allergic diseases in children [20], a study oppositely showed no difference between adolescents with different gender in the prevalence of eczema [36]. Results in the current study similarly manifested the relationship between higher dietary intake of Zn and lower odds of childhood eczema in children regardless of gender. Hormones significantly influence the pathogenesis of inflammatory diseases, such as asthma, rhinitis, and eczema [37]. Gender-related difference in asthma persistence may due to gender-related difference in susceptibility to oxidative stress, and the levels of 8-oxodG/creatinine was slightly higher in female gender [25]. However, the specific mechanisms of the gender difference between dietary antioxidants intake and childhood eczema needs further exploration.

The study population were from the NHANES database that were relatively representative for the population in the USA. We explored the association between different dietary antioxidants intake and childhood eczema, which may provide some information for future studies to clarify the roles of antioxidants on eczema, and thus may help to develop dietary prevention strategies for childhood eczema. However, there are some limitations in our study. Our study is an across-sectional study that was difficult to determine causal association and had inevitable biases. Information of dietary antioxidants intake was collected using the 24-h recalls of face-to-face interviews, which could only represent short-term dietary intake. Also, the prevalence of eczema may be underestimated by self-reported physician diagnoses. In addition, information of dietary antioxidants supplement, the family history of allergic diseases, vegetable and fruit allergy, antioxidants came from mother’s milk, and the subtype of eczema in the NHANES was not available. Therefore, further prospective cohort studies are warranted to explore the roles of different dietary antioxidants in childhood eczema in the future.

## Conclusion

Zn consumption was negatively associated with childhood eczema, and the roles of dietary antioxidants in childhood eczema are still needed further exploration.

### Supplementary Information

**Additional file 1: Table S1.** Covariates associated with childhood eczema. **Table S2. **Sensitivity analysis of characteristics before and after interpolation of missing data

## Data Availability

The datasets generated and/or analyzed during the current study are available in the NHANES database, https://wwwn.cdc.gov/nchs/nhanes/.

## Abbreviations

OS: Oxidative stress
ROS: Reactive oxygen species
Se: Selenium
Zn: Zinc
NHANES: National Health and Nutrition Examination Survey
MEC: Mobile Examination Center
PIR: Poverty-to-income ratio
BMI: Body mass index
CRP: C-reactive protein
IgE: Immunoglobulin E
PUFA: Polyunsaturated fatty acids
Mean ± SD: Mean ± standard deviation
ORs: Odds ratios
CIs: Confidence intervals
