# Supplementary material for: Association between dietary antioxidants intake and childhood eczema: results from the NHANES database
Source: J Health Popul Nutr. 2024 Jan 18;43:12. doi: 10.1186/s41043-024-00501-x (PMC10797884; doi:10.1186/s41043-024-00501-x)
Supplement: Supplementary file 1 — Additional file 1: Table S1. Covariates associated with childhood eczema. Table S2. Sensitivity analysis of characteristics before and after interpolation of missing data [file 41043_2024_501_MOESM1_ESM.docx]

**Table S1. Covariates associated with childhood eczema**

| Variables | OR (95% CI) | *P* |
| --- | --- | --- |
| Age | 0.95 (0.92-0.98) | <0.001 |
| Gender |  |  |
| Female | Ref |  |
| Male | 1.06 (0.82-1.37) | 0.633 |
| Race |  |  |
| Mexican American | Ref |  |
| Other Hispanic | 4.45 (2.17-9.13) | <0.001 |
| Non-Hispanic White | 4.04 (2.62-6.22) | <0.001 |
| Non-Hispanic Black | 5.22 (3.43-7.94) | <0.001 |
| Other races | 3.43 (1.81-6.51) | <0.001 |
| Family educational background |  |  |
| Less than 9th grade | Ref |  |
| 9-11th grade | 4.03 (1.67-9.69) | 0.002 |
| High school grade | 5.55 (2.37-13.02) | <0.001 |
| Some college | 6.81 (2.94-15.77) | <0.001 |
| College graduate | 10.58 (4.55-24.63) | <0.001 |
| PIR | 1.21 (1.12-1.32) | <0.001 |
| Exposure to environmental tobacco smoke |  |  |
| No | Ref |  |
| Yes | 0.99 (0.70-1.41) | 0.968 |
| Insurance |  |  |
| No | Ref |  |
| Yes | 2.91 (1.73-4.90) | <0.001 |
| Maternal age | 1.01 (0.99-1.04) | 0.174 |
| Asthma |  |  |
| No | Ref |  |
| Yes | 2.32 (1.74-3.10) | <0.001 |
| Hay fever |  |  |
| No | Ref |  |
| Yes | 3.13 (1.92-5.11) | <0.001 |
| Food allergy |  |  |
| No | Ref |  |
| Yes | 1.51 (1.15-1.99) | 0.003 |
| BMI | 0.97 (0.94-0.99) | 0.006 |
| Cotinine | 1.00 (0.99-1.00) | 0.422 |
| CRP | 0.89 (0.68-1.17) | 0.408 |
| Vitamin D | 1.00 (1.00-1.01) | 0.450 |
| Energy | 1.00 (1.00-1.00) | 0.167 |
| PUFA | 1.00 (0.98-1.01) | 0.600 |
| IgE | 1.01 (1.01-1.01) | 0.003 |

OR: odds ratio, CI: confidence interval, Ref: reference, PIR: poverty income ratio, BMI: body mass index, CRP: C-reactive protein, PUFA: polyunsaturated fatty acid, IgE: immunoglobulin E

**Table S2. Sensitivity analysis of characteristics before and after interpolation of missing data**

| Variables | After  interpolation  (n=2304) | Before  interpolation  (n=2304) | *P* |
| --- | --- | --- | --- |
| Vitamin D, nmol/L, Mean ± SD | 58.53 ± 18.62 | 58.54 ± 18.61 | 0.975 |
| CRP, mg/dL, M (Q_1_, Q_3_) | 0.04 (0.01, 0.13) | 0.04 (0.01, 0.13) | 0.947 |
| Peanut IgE, kU/L, M (Q_1_, Q_3_) | 0.25 (0.25, 0.25) | 0.25 (0.25, 0.25) | 0.983 |
| Egg IgE, kU/L, M (Q_1_, Q_3_) | 0.25 (0.25, 0.25) | 0.25 (0.25, 0.25) | 0.983 |
| Milk IgE, kU/L, M (Q_1_, Q_3_) | 0.25 (0.25, 0.25) | 0.25 (0.25, 0.25) | 0.937 |
| Shrimp IgE, kU/L, M (Q_1_, Q_3_) | 0.25 (0.25, 0.25) | 0.25 (0.25, 0.25) | 0.259 |
| Weight, kg, M (Q_1_, Q_3_) | 48.05 (28.40, 63.00) | 48.00 (28.40, 63.00) | 0.978 |
| Height (cm), Mean ± SD | 147.28 ± 23.04 | 147.29 ± 23.05 | 0.985 |
| BMI, kg/m^2^, Mean ± SD | 21.10 ± 6.02 | 21.09 ± 5.98 | 0.951 |
| PIR, M (Q_1_, Q_3_) | 1.82 (0.95, 3.50) | 1.81 (0.95, 3.50) | 0.963 |
| Maternal age, years, M (Q_1_, Q_3_) | 25.90 ± 6.00 | 25.93 ± 5.98 | 0.880 |

PIR: poverty income ratio, CRP: C-reactive protein, PUFA: polyunsaturated fatty acid, IgE: immunoglobulin E

Statistics: t test and rank sum test
